# Draft Genome Sequence of *Campylobacter jejuni* 11168H

**DOI:** 10.1128/genomeA.01556-16

**Published:** 2017-02-02

**Authors:** Sarah E. Macdonald, Ozan Gundogdu, Nick Dorrell, Brendan W. Wren, Damer Blake, Richard Stabler

**Affiliations:** a Pathology and Pathogen Biology, the Royal Veterinary College, Hatfield, Hertfordshire, United Kingdom; b Faculty of Infectious and Tropical Diseases, London School of Hygiene and Tropical Medicine, London, United Kingdom

## Abstract

*Campylobacter jejuni* is the most prevalent cause of food-borne gastroenteritis in the developed world. The reference and original sequenced strain *C. jejuni* NCTC11168 has low levels of motility compared to clinical isolates. Here, we describe the draft genome of the laboratory derived hypermotile variant named 11168H.

## GENOME ANNOUNCEMENT

*Campylobacter jejuni* is a Gram-negative, microaerophilic, spiral-shaped enteric pathogenic bacterium and is the leading cause of bacterial food-borne gastroenteritis worldwide (1). *C. jejuni* infection is associated with mild diarrhea to severe inflammatory enteritis. In most cases, *C. jejuni* infection is self-limiting, however, there can be life-threatening postinfection complications such as Guillain-Barré syndrome, an acute autoimmune paralyzing neuropathy (2). *C. jejuni* 11168H is a hypermotile clonal derivative of NCTC 11168 (3, 4). The motility of the original sequenced strain *C. jejuni* 11168 was noted to be significantly lower than that of fresh clinical isolates (4). However, it was noted that there was variable motility ranging from almost nonmotile to hypermotile and that this could readily derive the wild-type parent strain (4). *C. jejuni* 11168H has been used in several studies including *C. jejuni* pathogenesis glycan analysis, colonization of chickens, *Galleria mellonella* larvae, responses to oxidative and aerobic stresses, and the investigation into outer membrane vesicles (5–9).

*C. jejuni* 11168H was sequenced using an Illumina MiSeq (2 × 151 bp) which generated 1,168,138 reads and 171,157,831 bp. MiSeq reads were polished using Trimmomatic (10) (v0.33). A draft genome was assembled using VelvetOptimiser (http://bioinformatics.net.au/software.velvetoptimiser.shtml). Assembled contigs were further polished using SSPACE (standard v3.0) (11), GapFiller (12) (v1.10), and Pilon (13) (v 1.16). Contigs were ordered with Abacas and Mauve and finally annotated using Prokka (14) (v1.11). The draft genome consisted of 83 contigs, totaling 1,615,620 bp with 30.5% G+C. Prokka identified 44 tRNAs and rRNAs, one clustered regularly interspaced short palindromic repeat (CRISPR), and 1,631 coding sequences (CDS).

### Accession number(s).

This whole-genome shotgun project has been deposited in the European Nucleotide Archive under the accession no. FPEE01000001 to FPEE01000083. The version described in this paper is the first version, FPEE01000000.
